# East Weddell Sea echinoids from the JR275 expedition

**DOI:** 10.3897/zookeys.504.8860

**Published:** 2015-05-18

**Authors:** Thomas Saucède, Huw Griffiths, Camille Moreau, Jennifer A. Jackson, Chester Sands, Rachel Downey, Adam Reed, Melanie Mackenzie, Paul Geissler, Katrin Linse

**Affiliations:** 1 UMR CNRS 6282 Biogéosciences, Université de Bourgogne, 6, bd Gabriel 21000, Dijon, France; 2 British Antarctic Survey (BAS), High Cross Madingley Road, CB3 0ET, Cambridge, United Kingdom; 3 Sektion Marine Evertebraten I, Forschungsinstitut und Naturmuseum Senckenberg, Frankfurt am Main, Germany; 4 School of Ocean and Earth Science, National Oceanography Centre Southampton, University of Southampton, United Kingdom; 5 Marine Science Department, Museum Victoria, Australia

**Keywords:** Echinoidea, Southern Ocean, Biodiversity

## Abstract

Information regarding the echinoids in this dataset is based on the Agassiz Trawl (AGT) and epibenthic sledge (EBS) samples collected during the British Antarctic Survey cruise JR275 on the RRS James Clark Ross in the austral summer 2012. A total of 56 (1 at the South Orkneys and 55 in the Eastern Weddell Sea) Agassiz Trawl and 18 (2 at the South Orkneys and 16 in the Eastern Weddell Sea) epibenthic sledge deployments were performed at depths ranging from ~280 to ~2060 m. This presents a unique collection for the Antarctic benthic biodiversity assessment of an important group of benthic invertebrates. In total 487 specimens belonging to six families, 15 genera, and 22 morphospecies were collected. The species richness per station varied between one and six. Total species richness represents 27% of the 82 echinoid species ever recorded in the Southern Ocean (David et al. 2005b, Pierrat et al. 2012, Saucède et al. 2014). The Cidaridae (sub-family Ctenocidarinae) and Schizasteridae are the two most speciose families in the dataset. They comprise seven and nine species respectively. This is illustrative of the overall pattern of echinoid diversity in the Southern Ocean where 65% of Antarctic species belong to the families Schizasteridae and Cidaridae (Pierrat et al. 2012).

## Project details

**Project title:** JR 275 RRS James Clark Ross 2012

**Personnel:** Huw Griffiths, Camille Moreau, Jennifer Jackson, Chester Sands, Rachel Downey, Adam Reed, Melanie Mackenzie, Paul Geissler, Katrin Linse

**Funding:** This study is part of the British Antarctic Survey Polar Science for Planet Earth Programme funded by the Natural Environment Research Council. Funding for T. Saucède to visit and identify material was provided by the vERSO program (Ecosystem Responses to global change: a multiscale approach in the Southern Ocean). This is contribution no. 3 to the vERSO project (www.versoproject.be), funded by the Belgian Science Policy Office (BELSPO, contract n°BR/132/A1/vERSO). This is a contribution to the SCAR (Scientific Committe on Antarctic Research) AntEco (State of the Antarctic Ecosystem) Programme.

**Study extent description:** The study area of this dataset was set in the Eastern Weddell Sea and focused on sampling the continental shelf, upper slope and over-deepened shelf basins of the Filchner Trough region of the Weddell Sea (Knust and Schröder 2014). This dataset presents species occurrences and species richness of the individual trawls (Agassiz Trawl and Epibenthic Sledge deployments). Our sampling regime was designed to investigate patterns of biodiversity, and once compared to other sources of material, biogeography and phylogeography in the benthos of this region of the Southern Ocean. The Filchner Trough region is an oceanographically interesting area that includes regions of cold Antarctic Bottom Water (ABW) production. One of the other characteristics of the area is the perennial sea ice cover and the presence of very large icebergs.

**Design description:** The South-Eastern Weddell Sea is a relatively under sampled area on the Antarctic continental shelf, according to a recent gap analysis carried out by Griffiths et al. (2011). EvolHist (Evolutionary History of the Polar Regions), a core project at the British Antarctic Survey, studied the South-Eastern Weddell Sea to assess the biodiversity at local and regional scales (comparable to the BIOPEARL 2006 cruise to the Scotia Sea and the BIOPEARL II 2008 cruise to the Bellingshausen and Amundsen Seas) and investigate the phylogenetic relationships of selected marine invertebrate taxa and their biogeography in reference to the climatological, oceanographical and geological history of the Weddell Sea. The results are used to determine of the role of Antarctica and extreme environments in general in evolutionary innovation and generation of global biodiversity. The species presence data are added to SOMBASE (South- ern Ocean Mollusc Database www.antarctica.ac.uk/sombase). SOMBASE generated a significant portion of the initial core data system upon which SCAR’s Antarctic Biodiversity Information Facility (AntaBIF, www.biodiversity.aq) was built. As AntaBIF (and its predecessor, SCAR-MarBIN) is the Antarctic Node of the international OBIS and GBIF networks, the SOMBASE data system was designed to comply with the Darwin Core standards. Regarding the dataset, the existing Data Toolkit from AntaBIF was used (http://ipt.biodiversity.aq/), following the OBIS schema (http://iobis.org/data/schema-and-metadata). The dataset was up- loaded in the ANTOBIS (Antarctic Ocean Biogeographic Information System) database (the geospatial component of SCAR-MarBIN), and the taxonomy was matched against the Register of Antarctic Marine Species, using the Taxon Match tool (http://www.scarmarbin.be/rams.php?p=match). The dataset meets the Darwin Core requirements and was designed around this data schema.

**Figure 1.**
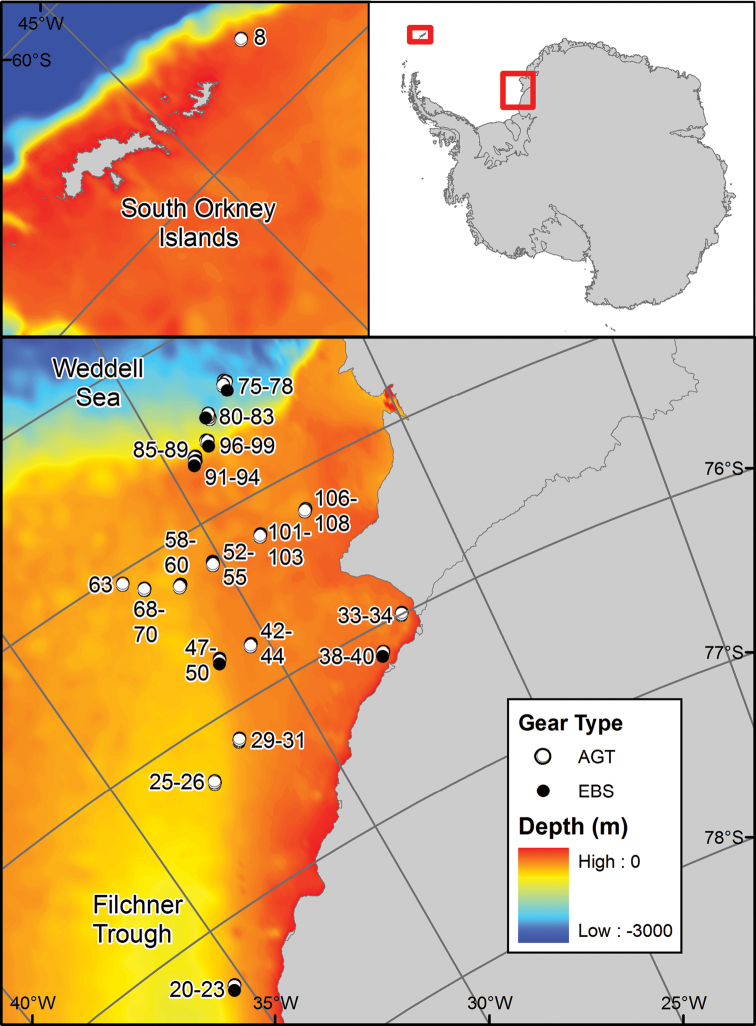
Sample locations for JR275 echinoid records.

**Sampling description:** A single test location off the South Orkney Islands and a further six locations in the Eastern Weddell Sea at different depths ranging from 279 to 2058m have been sampled using an Agassiz Trawl (AGT) and an epibenthic sledge (EBS). Most of the Weddell Sea deployments were made along two transects, one running from south to north along the edge of the Filchner Trough and one running from west to east out of the Filchner Trough onto the shallower shelf. Two further localities in overdeepened basins close to the Brunt Ice shelf were sampled (Figure 1, Stations 33-40). At each site, three replicate Agassiz trawls (individual stations) were taken and where the substrate was suitable (not too rocky) a single EBS deployment was conducted. The JR275 cruise report is available from the British Oceanographic Data Centre (www.bodc.ac.uk/data/information_and_inventories/cruise_inventory/report/10598).

This dataset represents 48 AGT and 8 EBS deployments: consisting of a single deployment at the South Orkneys at 279m; 15 at depths of ~400m; four at ~500m; 21 at ~600m; two at ~700m and four deployments at each of ~1000m, ~1500m and ~2000m deep (Figure 1, Table 1).

**Table 1.** Sampling stations containing echinoid samples from JR275. AGT = Agassiz Trawl, EBS = Epibenthic sledge.

Station ID	Gear type	Start lat	End lat	Start long	End long	Min depth	Max depth	Date
8	AGT	-60.6774	-60.6775	-44.01327	-44.0144	279.04	281.57	12/02/2012
20	AGT	-77.359	-77.3576	-35.37029	-35.3642	654.34	654.35	19/02/2012
21	AGT	-77.3548	-77.3529	-35.35131	-35.3423	648.18	652.8	19/02/2012
23	EBS	-77.3569	-77.3579	-35.36059	-35.365	649.74	655.86	19/02/2012
25	AGT	-76.3295	-76.327	-32.90046	-32.8956	778.81	781.73	20/02/2012
26	AGT	-76.321	-76.3197	-32.88435	-32.8819	780.3	789.24	20/02/2012
29	AGT	-76.1991	-76.1982	-31.86015	-31.8556	575.95	578.97	20/02/2012
30	AGT	-76.1956	-76.1947	-31.84258	-31.8383	575.99	578.94	20/02/2012
31	AGT	-76.1919	-76.191	-31.82427	-31.8197	564.11	573	20/02/2012
33	AGT	-76.0231	-76.0222	-26.99542	-26.9909	605.21	610	21/02/2012
34	AGT	-76.0196	-76.0187	-26.97793	-26.9735	608	613	21/02/2012
38	AGT	-76.1697	-76.1685	-27.79567	-27.799	544.89	561	21/02/2012
39	AGT	-76.1694	-76.1689	-27.79659	-27.798	549.28	555.26	21/02/2012
40	EBS	-76.1669	-76.1657	-27.8038	-27.8073	533.05	550.82	21/02/2012
42	AGT	-75.7612	-75.7621	-30.43723	-30.4413	429.41	433.85	22/02/2012
43	AGT	-75.7645	-75.765	-30.45297	-30.4547	427.94	430	22/02/2012
44	AGT	-75.767	-75.7674	-30.46317	-30.4648	429.39	436.8	22/02/2012
47	AGT	-75.7406	-75.7418	-31.23803	-31.2413	578.94	584.88	22/02/2012
48	AGT	-75.7451	-75.7462	-31.25064	-31.2538	584.83	590.75	22/02/2012
49	AGT	-75.7496	-75.7508	-31.2636	-31.2668	583.36	584.94	22/02/2012
50	EBS	-75.7433	-75.7459	-31.24615	-31.2535	583.34	590.45	22/02/2012
52	AGT	-75.2434	-75.2447	-30.24534	-30.2472	418.73	419.21	23/02/2012
53	AGT	-75.2478	-75.2491	-30.25152	-30.2533	417.39	417.78	23/02/2012
54	AGT	-75.2526	-75.2539	-30.25835	-30.2602	418.7	419.11	23/02/2012
55	AGT	-75.2567	-75.258	-30.26436	-30.2662	418.38	418.61	23/02/2012
58	AGT	-75.2631	-75.2638	-31.12627	-31.131	604.29	607.13	23/02/2012
59	AGT	-75.2658	-75.2665	-31.14481	-31.1504	607.1	610.24	23/02/2012
60	AGT	-75.2686	-75.2692	-31.16355	-31.168	614.3	616.52	23/02/2012
63	AGT	-75.0852	-75.0866	-32.21766	-32.2177	609.48	612.28	24/02/2012
68	AGT	-75.1767	-75.1781	-31.8702	-31.869	655.78	676.11	24/02/2012
69	AGT	-75.1754	-75.1768	-31.87114	-31.87	654.87	657.46	24/02/2012
70	AGT	-75.1743	-75.1757	-31.87206	-31.8708	654.65	691.31	24/02/2012
75	AGT	-74.37	-74.3718	-28.10797	-28.1	2052.26	2053.91	26/02/2012
76	AGT	-74.3797	-74.3817	-28.06634	-28.059	2056.14	2058.19	26/02/2012
77	AGT	-74.3886	-74.3904	-28.1561	-28.1482	2006.54	2011.16	26/02/2012
78	EBS	-74.4047	-74.4065	-28.08486	-28.0769	2019.49	2026.16	26/02/2012
80	AGT	-74.5202	-74.5175	-28.75306	-28.7512	1537.72	1545.99	28/02/2012
81	AGT	-74.5084	-74.5057	-28.74527	-28.7436	1558.28	1570.08	28/02/2012
82	AGT	-74.4962	-74.4931	-28.73726	-28.7352	1580.27	1595.46	28/02/2012
83	EBS	-74.4853	-74.4846	-28.77472	-28.7847	1577.88	1588.23	28/02/2012
85	AGT	-74.6741	-74.675	-29.42462	-29.4344	586.74	604.49	29/02/2012
86	AGT	-74.6769	-74.6766	-29.45447	-29.4507	573.42	580.99	29/02/2012
88	AGT	-74.6747	-74.6745	-29.43061	-29.4284	592.71	602.27	29/02/2012
89	EBS	-74.6716	-74.6706	-29.39886	-29.3883	639.32	657.44	29/02/2012
91	AGT	-74.7067	-74.7054	-29.50822	-29.5066	401.67	410	29/02/2012
92	AGT	-74.7013	-74.7009	-29.50091	-29.5002	427.17	428.55	29/02/2012
93	AGT	-74.6982	-74.6975	-29.49652	-29.4956	439.76	450.09	29/02/2012
94	EBS	-74.6919	-74.6893	-29.48786	-29.4842	476.94	494.03	29/02/2012
96	AGT	-74.6252	-74.6268	-29.05155	-29.0429	1018.91	1028.48	01/03/2012
97	AGT	-74.6304	-74.6319	-29.0236	-29.0151	985.75	1010.63	01/03/2012
99	EBS	-74.6341	-74.6357	-29.00812	-28.9996	958.98	986.19	01/03/2012
101	AGT	-75.2427	-75.2437	-29.00356	-29.0072	391.66	398.3	04/03/2012
102	AGT	-75.246	-75.2471	-29.01541	-29.019	392.77	396.83	04/03/2012
103	AGT	-75.2495	-75.2506	-29.02708	-29.0304	390.17	392.2	04/03/2012
106	AGT	-75.2389	-75.2397	-27.84859	-27.853	413.67	415.71	04/03/2012
108	AGT	-75.244	-75.2448	-27.87707	-27.8816	417.56	424.41	04/03/2012

The AGT had an inner mesh size of 1 cm and a mouth width of 2 m. The EBS consisted of an epi-(below) and a supra-(above) net. Each of these nets has a mesh size of 500µm and an opening of 100×33cm. The cod end of both nets is equipped with net-buckets containing a 300µm mesh window (Brenke 2005). The AGT and EBS were trawled for 10 minutes (depending on depth, seabed type and the condition of the animals in the initial trawl) on the sea bed at a 1 knot speed. Following Brenke (2005), since the EBS epi- and supra-nets collect the same fauna, they were pooled and treated as a single sample.

**Quality control description:** A species name was given to each specimen when it was possible. Identifications and taxonomic accuracies are based on David et al. (2005a, 2005b), Pierrat et al. (2012), and Saucède et al. (2014). When identification was inconclusive, e.g. for small specimens at very early stages of development, only family or genus names were assigned. These specimens were referred to as gen. *sp.* or genus name *sp.* respectively and might belong to one of the species listed in the dataset (Table 2). Specimens referred to as *Abatus sp.* 1 belong to none of the species listed in the dataset. The specimen referred to in the dataset as Amphipneustes aff. similis is very similar in morphology to *Amphipneustes similis* but it presents distinctive morphological characters that are not diagnostic of the aforementioned species. While included in this dataset as Amphipneustes aff. similis it is likely that this will be described as a new species after further morphological and genetic analyses.

**Table 2.** Presence only matrix of echinoid species from JR275.

			Station Number																																																							
Family	Genus	Species	8	20	21	23	25	26	29	30	31	33	34	38	39	40	42	43	44	47	48	49	50	52	53	54	55	58	59	60	63	68	69	70	75	76	77	78	80	81	82	83	85	86	88	89	91	92	93	94	96	97	99	101	102	103	106	108
Cidaridae	*Aporocidaris*	*milleri*																																	**X**																							
	*Ctenocidaris*	*gigantea*																																									**X**															
		*perrieri*																																									**X**													**X**		
	*gen.*	*sp.*																																			**X**									**X**				**X**								
	*Notocidaris*	*gaussensis*																																	**X**	**X**					**X**																	
		*lanceolata*													**X**																																											
		*mortenseni*																																									**X**		**X**													
	*Rhynchocidaris*	*triplopora*	**X**																																								**X**				**X**	**X**	**X**									
Echinidae	*Sterechinus*	*antarcticus*		**X**	**X**				**X**	**X**	**X**			**X**						**X**	**X**	**X**						**X**	**X**	**X**	**X**		**X**	**X**									**X**	**X**	**X**			**X**							**X**			**X**
		*dentifer*																																	**X**		**X**		**X**	**X**	**X**																	
		*sp.*				**X**	**X**	**X**				**X**	**X**			**X**									**X**		**X**					**X**						**X**				**X**				**X**				**X**		**X**	**X**					
Plexechinidae	*Plexechinus*	*planus*																																							**X**																	
Pourtalesiidae	*Pourtalesia*	*hispida*																																		**X**	**X**				**X**																	
		*Sp.*																																				**X**																				
Schizasteridae	*Abatus*	*sp.* 1																																												**X**					**X**							
	*Amphipneustes*	aff. *similis*																				**X**																																				
		*lorioli*																**X**						**X**						**X**																									**X**	**X**	**X**	**X**
		*similis*																		**X**				**X**		**X**		**X**																	**X**													
	*Brachysternaster*	*chesheri*									**X**						**X**	**X**						**X**											**X**																							
	*Delopatagus*	*brucei*																																		**X**																						
	*gen.*	*sp.*																					**X**																											**X**				**X**			**X**	
	*Tripylaster*	*philippii*																																							**X**																	
	*Tripylus*	*abatoides*																																									**X**															
		*cordatus*																	**X**																																							
Urechinidae	*Antrechinus*	*nordenskjoldi*																																									**X**			**X**							**X**					

This dataset presents species occurrences and species richness of the individual AGT and EBS deployments.

## Taxonomic coverage

**General taxonomic coverage description:** The present dataset focuses on the class Echinoidea (Echinodermata). It includes six families, 15 genera, and 22 species:

**Class:** Echinoidea

**Family:** Cidaridae, Echinidae, Plexechinidae, Pourtalesiidae, Schizasteridae, Urechinidae

**Genus:** *Aporocidaris*, *Ctenocidaris*, *Notocidaris*, *Rhynchocidaris*, *Sterechinus*, *Plexechinus*, *Pourtalesia*, *Abatus*, *Amphipneustes*, *Brachysternaster*, *Delopatagus*, *Tripylaster*, *Tripylus*, *Antrechinus*, *Cystechinus*

**Species:** *Aporocidaris milleri*, *Ctenocidaris gigantea*, *Ctenocidaris perrieri*, *Notocidaris gaussensis*, *Notocidaris lanceolata*, *Notocidaris mortenseni*, *Rhynchocidaris triplopora*, *Sterechinus antarcticus*, *Sterechinus dentifer*, *Plexechinus planus*, *Pourtalesia hispida*, *Abatus sp.* 1, Amphipneustes aff. similis, *Amphipneustes lorioli*, *Amphipneustes similis*, *Brachysternaster chesheri*, *Delopatagus brucei*, *Tripylaster philippii*, *Tripylus abatoides*, *Tripylus cordatus*, *Antrechinus nordenskjoldi*, *Cystechinus wyvillii*

## Spatial coverage

**General spatial coverage:** East Weddell Sea, Antarctica

**Coordinates:** 60.68°S and 77.36°S; 44.01°W and 26.78°W

**Temporal coverage:** February 12, 2012–March 4, 2012

## Natural collections description

**Parent collection identifier:** British Antarctic Survey **Collection name:** EvolHist Weddell Sea Echinoids

**Collection identifier:** Saucède

**Specimen preservation method:** Ethanol

## Methods

**Method step description:**

- Agassiz trawl sampling in the Weddell Sea
- Once on board, the samples were photographed as total catch and then hand-sorted into groups varying from Phylum to species level collections. Representatives of many taxa were photographed in detail. The wet‐mass (biomass) of the different taxa was assessed by using calibrated scales (with accuracy and resolution of 0.001 kg). Samples were fixed in 96% undenatured and precooled (at -20°C) ethanol (Linse 2008) and kept for a minimum of 48 hours in a -20°C freezer, with rotation of containers to ensure full preservation of material.
- Epibenthic sledge sampling in the Weddell Sea
- Once on the deck, the content of the samplers from the first deployment was immediately fixed in 96% undenatured and precooled (at -20°C) ethanol and kept for a minimum of 48 hours in a -20°C freezer.
- The taxonomic identification was performed in the British Antarctic Survey laboratory using a stereomicroscope.

## Datasets

**Dataset description**

**Object name: BAS_JR275_Echinoidea**

**Character encoding:** UTF-8

**Format name:** Darwin Core Archive format

**Format version:** 1.0

**Distribution:** http://ipt.biodiversity.aq/resource.do?r=bas_jr275_echinoidea

**Publication date of data:** 27/10/2014

**Language:** English

**Metadata language:** English

**Date of metadata creation:** 27/10/2014

**Hierarchy level:** Dataset
